# Vertical GaN‐On‐GaN Micro‐LEDs for Near‐Eye Displays

**DOI:** 10.1002/advs.202506784

**Published:** 2025-07-30

**Authors:** Zichun Li, Yibo Liu, Haonan Jiang, Feng Feng, Jingyang Zhang, Shan Huang, Fion Yeung, Manchun Tseng, Man Wong, Hoi Sing Kwok, Zhaojun Liu

**Affiliations:** ^1^ Hong Kong University of Science and Technology Hong Kong 999077 China; ^2^ Southern University of Science and Technology Shenzhen 518055 China

**Keywords:** GaN‐on‐GaN, ion implantation, micro‐LEDs, vertical structure

## Abstract

In various micro‐light‐emitting diode (micro‐LED) display products, near‐eye applications such as AR (augmented reality) and VR (virtual reality) are gaining popularity, driving consumer demand for higher brightness, resolution, and compact size. To address more advanced demands, GaN‐on‐GaN homoepitaxial micro‐LEDs are notable for their low defect density, excellent thermal management, high efficiency, etc. Additionally, the conductivity of the GaN substrate enables the efficient integration of vertical micro‐LEDs, further enhancing performance for near‐eye displays. In this work, GaN‐on‐GaN homoepitaxial platforms to fabricate low‐defect‐density micro‐LEDs are leveraged with superior electrical properties, addressing the limitations of conventional heterogeneous substrates. By replacing traditional ICP (Inductively coupled plasma) mesa etching with fluorine ion implantation for pixel isolation, this study achieves significant reductions in series resistance and enhances optical performance, characterized by sharper pixel edges and a narrowed full width at half maximum (FWHM). Furthermore, the implementation of vertical micro‐LED architectures enables a compact device footprint, facilitating ultra‐dense integration for near‐eye systems. To evaluate performance under practical operating conditions, the effective external quantum efficiency (EQE_effective_) is introduced. The ion‐implanted vertical structures demonstrate a substantial improvement in EQE_effective_ over traditional ICP‐etched devices, underscoring their potential for high‐brightness applications. This work advances high‐resolution, energy‐efficient micro‐LED technologies, offering a scalable pathway for next‐generation AR/VR displays.

## Graphical abstract

This work advances micro‐light‐emitting diode (micro‐LED) technology using GaN‐on‐GaN homoepitaxial platforms for low defects. Fluorine ion implantation replaces plasma etching for pixel isolation, reducing series resistance and enhancing optical quality. Vertical architectures enable dense integration. Introduced display effective external quantum efficiency shows gains for high‐brightness augmented/virtual reality applications, offering scalability.

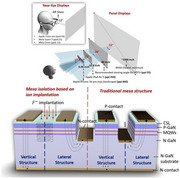

## 1 Introduction

**Figure 1:**
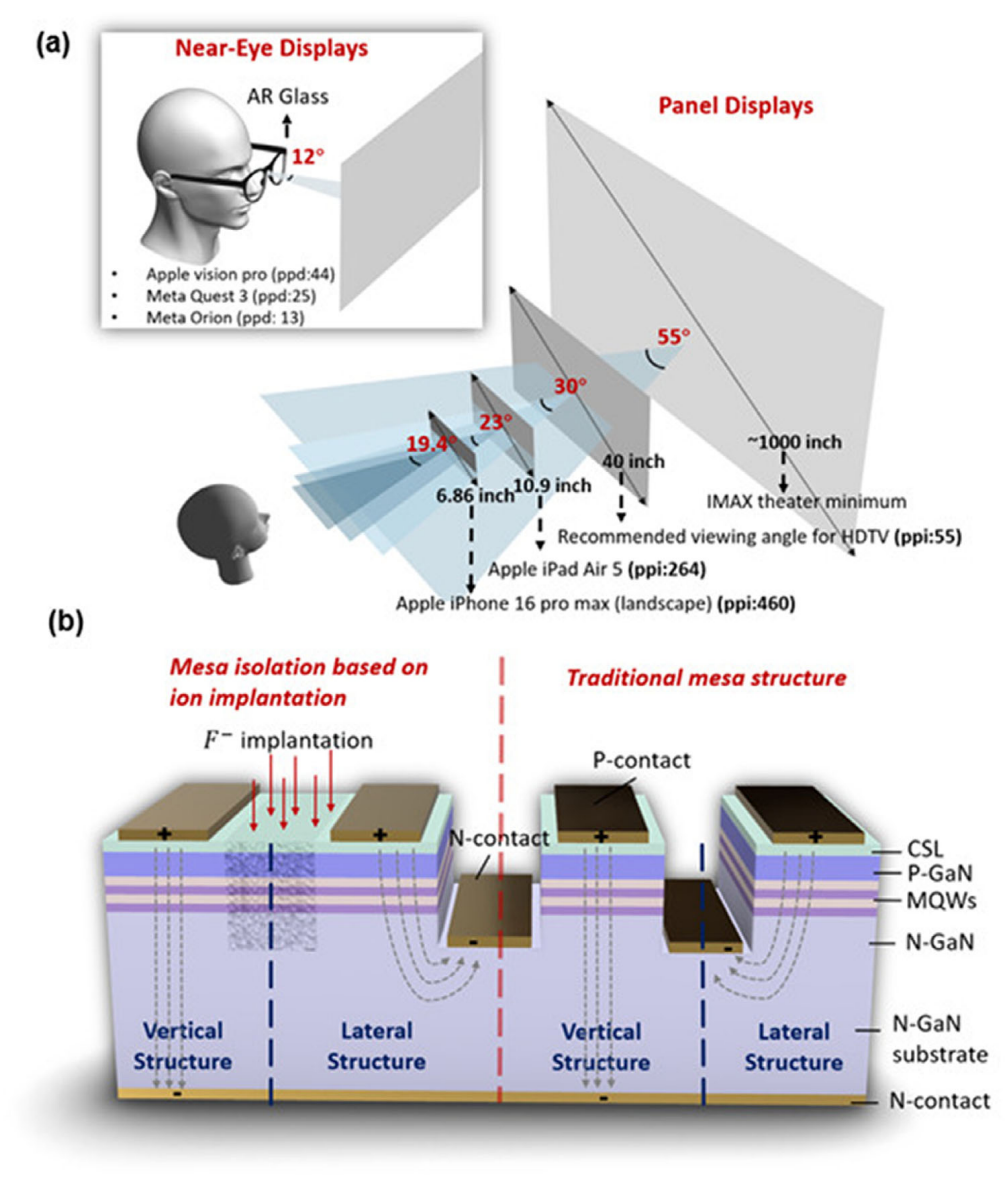
a) The demand for horizontal FOV for display products of different sizes (inches) and PPI for panel display products, PPD for near‐eye display products. The FOV data is sourced from Professor Thad Starner's presentation at Photonics West 2019 (PW2019). b) The four types of micro‐LED device structures used in this work, from left to right: vertical and lateral structures based on ion implantation (F^‐^) for pixel isolation, and vertical and lateral structures based on traditional mesa etching (the passivation layer is not shown in the figure). The grey dashed line indicates the direction of current.

Our daily lives are inseparable from various display products, which can be roughly divided into two categories. One category is near‐eye displays based on waveguides, such as augmented reality (AR) and virtual reality (VR), while the other category is panel displays based on mass transfer, such as smartphone and television screens, as shown in **Figure** **1**a. Different sizes of display products have different fields of view (FOV). Many manufacturers are currently trying to maximize FOV. However, based on the usage patterns of these products in everyday life, a large FOV is often unnecessary. In most instances, users engage with display devices from a direct viewing angle, typically at a limited angle, particularly in the case of near‐eye applications such as smartphones and AR glasses, which are designed for shorter viewing distances. The demand for FOV (in the horizontal direction) for different products is also shown in Figure 1a. The view angle decreases as the view distance of the screen decreases. For near‐eye display applications, a 12° FOV is enough. Given that the current FOV meets the requirements, efforts should be made to enhance the axial display brightness and display quality when viewed directly.

With the continuous advancement in display technology, gallium nitride (GaN)‐based micro‐light‐emitting diodes (micro‐LEDs) have gained widespread application due to their high contrast, long lifespan, and excellent stability, particularly in near‐eye display fields.^[^ ^1^, ^2^, ^3^, ^4^, ^5^, ^6^, ^7^ ^]^ Currently, most GaN‐based micro‐LEDs use sapphire as the substrate and employ Inductively Coupled Plasma (ICP) etching to achieve mesa structures for pixelization.^[^ ^8^ ^]^ However, several challenges still slow down their development.

On the one hand, the high‐energy ion bombardment introduced during the ICP etching process can create sidewall defects, such as gallium dangling bonds, resulting in defect energy levels within the multi‐quantum well (MQW) active region.^[^ ^9^, ^10^ ^]^ This significantly increases Shockley–Read–Hall (SRH) non‐radiative recombination, ultimately reducing light emission efficiency. As device sizes decrease, the proportion of sidewall area increases, making these issues worse.^[^ ^11^ ^]^ Moreover, the light emitted from the sidewalls is largely unhelpful for near‐eye display applications and may even be harmful. As discussed earlier, in most scenarios, our line of sight is directed forward, and at this time, side‐emitted light is ineffective for the overall display effect. More critically, this side emission can adversely affect adjacent pixels, leading to optical crosstalk and diminishing display contrast.^[^ ^12^, ^13^ ^]^ On the other hand, heteroepitaxial substrates such as sapphire present issues related to lattice mismatch and thermal mismatch, which can make the quantum‐confined Stark effect (QCSE) worse.^[^ ^14^ ^]^ This phenomenon leads to a shift in the central wavelength, negatively impacting the performance and efficiency of the devices. Additionally, conventional sapphire substrates (n≈1.77) propagate photons between pixels via waveguiding effects, causing significant substrate‐mediated crosstalk.^[^ ^15^ ^]^ Therefore, there is an urgent need for a more comprehensive solution to meet the growing demand for near‐eye micro‐displays.

For near‐eye micro‐LED display applications, achieving higher brightness and resolution, as well as smaller sizes, is essential. To enhance brightness and resolution, we need to address the issues caused by the sidewalls. Surface defect‐induced non‐radiative recombination can be reduced through methods such as sidewall passivation,^[^ ^16^, ^17^ ^]^ wet chemical treatment,^[^ ^18^ ^]^ and thermal annealing.^[^ ^19^ ^]^ Another approach is to eliminate sidewalls by using ion implantation for pixel isolation. This technique involves injecting specific ions into designated areas, causing lattice damage and reduced conductivity, which achieves electrical isolation and addresses issues related to sidewall emissions. Recent advances confirm its efficacy: an N⁺ implantation study on Si substrates achieved 33% external quantum effeciency (EQE) gain through sidewall passivation^[^ ^20^ ^]^; Ye et al. further suppressed leakage currents to 1.45 × 10^‐^⁷ A (at −5 V) in 10 µm pixels via controlled F^‐^ energy/dose (90–180 keV, 1 × 10^15^ ions cm^−^^2^) on sapphire^[^ ^21^ ^]^; while Xu et al. demonstrated 82.1 W cm^−^^2^ output for vertical µLEDs but noted <10 µm efficiency limits.^[^ ^22^, ^23^ ^]^ Additionally, the ion implantation pixel isolation method has been proven to reduce optical crosstalk.^[^ ^24^, ^25^ ^]^ By creating a planar isolation barrier with a refractive index closely matched to GaN (≈2.3 versus 2.4), ion implantation minimizes interfacial light scattering and reflection, effectively confining emitted photons within the intended pixel and suppressing the lateral escape modes responsible for inter‐pixel glow. Despite these advances, these approaches remain constrained by heteroepitaxial substrate limitations, including thermal management challenges and defect‐induced efficiency degradation. GaN homoepitaxial substrates eliminate heterointerface lattice mismatch, suppressing non‐radiative recombination through a three‐order‐of‐magnitude reduction in threading dislocation density (TDD) – from 4.34 × 10^8^ cm^‐^^2^ (GaN‐on‐sapphire) to 7.09 × 10^5^ cm^‐^^2^ (GaN‐on‐GaN) as determined by X‐ray diffraction rocking curve analysis and corroborated by cathodoluminescence mapping (≈10^8^→≈10^5^ cm^‐^^2^) in our prior work,^[^ ^1^, ^5^, ^26^ ^]^– thereby fundamentally enhancing radiative recombination efficiency.

To achieve smaller size, the electrode configuration is adjusted from a lateral structure (with electrodes on the same side of the substrate) to a vertical structure (with electrodes on opposite sides). Conventional verticalization relies heavily on transfer techniques,^[^ ^27^, ^28^, ^29^ ^]^—Tian et al. demonstrated flexible vertical µLEDs by bonding InGaN epilayers to AuSn substrates via laser lift‐off (LLO), achieving 40 MHz bandwidth.^[^ ^30^ ^]^ Subsequent substrate thinning via mechanical polishing or chemical mechanical polishing (CMP) reduces thickness for current spreading but introduces yield loss and stress‐induced defects.^[^ ^31^ ^]^ Despite these efforts, no prior work has directly utilized conductive GaN homoepitaxial substrates for native GaN‐on‐GaN vertical µLEDs. By leveraging GaN's inherent advantages—high conductivity, transparency, and thermal conductivity —our approach eliminates transfer/thinning steps. This fundamentally reduces process complexity, minimizes pixel area, eradicates current crowding, and enhances thermal management threefold compared to sapphire‐based vertical structures.

In this study, we present an advanced approach to GaN‐based micro‐LEDs for near‐eye microdisplays by integrating ion‐implanted pixel isolation and vertical device architectures on GaN homoepitaxial substrates. Unlike conventional mesa etching, the ion implantation method significantly reduces series resistance and optical crosstalk, enabling sharper pixel definition and enhanced luminous efficiency. Combined with vertical structures, this approach not only maintains consistent performance at high current densities but also facilitates smaller pixel pitches, addressing the critical demand for higher resolution in AR/VR applications. Our results demonstrate the potential of this synergistic strategy to advance micro‐LED technology for next‐generation near‐eye displays.

## 2 Experimental Section

The GaN‐based Micro‐LED epitaxial structure was grown on a 2‐inch free‐standing GaN substrate via metalorganic chemical vapor deposition (MOCVD). The homoepitaxial layers were sequentially deposited as follows: a 1800 nm Si‐doped n‐GaN layer, a 30 nm Si‐doped n‐Al_0.05_Ga_0.95_N electron spreading layer (ESL), a 200 nm n‐GaN spacer, a stress‐relaxation layer (SRL) consisting of 3 periods of u‐In_0.05_GaN (1 nm)/n‐GaN (49 nm), a 10‐period MQW active region with u‐In_x_GaN (3 nm)/n‐GaN (12 nm) (x = 0.15), a 20 nm p‐Al_0.05_Ga_0.95_N electron blocking layer, and a 60 nm Mg‐doped high‐temperature (HT) p‐GaN layer. To facilitate the subsequent fabrication of vertical structures, double‐side polishing was performed. A 100 nm indium tin oxide (ITO) layer was deposited on p‐GaN as a current spreading layer (CSL) using electron beam technology, followed by rapid thermal annealing to achieve ohmic contact between ITO and p‐GaN. Next, the mesa structure was defined using photolithography with a 500 nm plasma‐enhanced chemical vapor deposition (PECVD) SiO_2_ layer as a hard mask, which also serves as a mask for subsequent ion implantation. ITO and p‐GaN were then etched using ICP. After that, half of the sample was covered with a silicon wafer, and the GaN was etched to obtain the traditional mesa structure, followed by treatment with an 8% KOH solution to reduce sidewall damage. Subsequently, the other half (the area already etched) was covered with a silicon wafer, and fluorine (F^−^) ion implantation was performed at an angle of 7°, with an energy of 150 keV and a dose of 1E15 (Simulation results are presented in Figure S1, Supporting Information). The use of F^−^ was due to their ability to suppress lateral dispersion while maintaining thermal stability, as these medium‐mass ions were injected.^[^ ^21^ ^]^ The formation of Ga‐F bonds allows for F^‐^ implantation to retain thermal stability even at temperatures up to 550 °C.^[^ ^32^ ^]^ Finally, a dual‐layer passivation film was deposited on the entire wafer, consisting of a 30 nm thermal atomic layer deposition (ALD) Al_2_O_3_ layer and a 300 nm PECVD SiO_2_ layer, to passivate the remaining dangling bonds on the sidewalls. 300 nm Ti/Al/Ni/Au electrodes were then evaporated on the ITO, as well as on the front and back of the n‐GaN.

At this point, four different structures were obtained on the same sample (as shown in Figure 1b): lateral and vertical structured devices obtained using the traditional mesa ICP etching method, and lateral and vertical structured devices obtained using the ion implantation method. It should be noted that the n‐electrode in the lateral structure uses the n‐electrode from the nearest adjacent mesa etched structure on the same sample.

## 3 Results and Discussion

### 3.1 Electrical Performance

**Figure 2:**
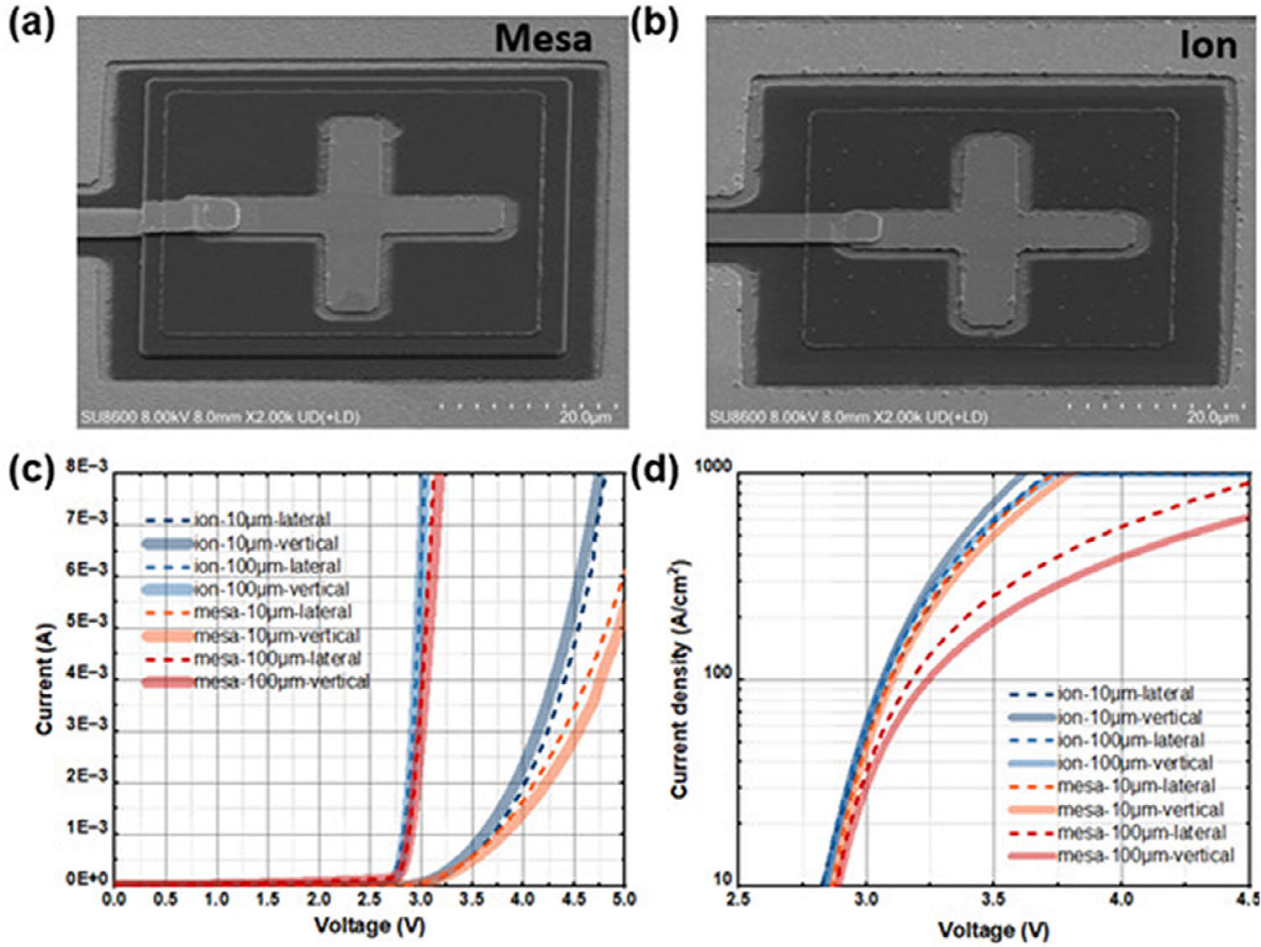
a,b) are SEM images of devices with a size of 50 × 50 µm^2^ taken at an angle of 50°. IV c) and JV d) curves of 100 × 100 µm^2^ and 10 × 10 µm^2^ devices with lateral and vertical structures using ion implantation and mesa etching.

In the figures and the following text, “mesa” refers to devices using the mesa ICP etching process, while “ion” or “ion implantation” refers to devices using the ion implantation process. **Figure** **2**a,b shows the SEM images of devices with a size of 50 × 50 µm^2^ that utilize mesa etching and ion implantation for pixel isolation, respectively. The cross shape on the surface represents the p‐electrode. In Figure 2a, a distinct step is observed, indicating that pixel isolation is achieved through the etching of the mesa structure. In contrast, Figure 2b shows a smoother surface at the corresponding position without etched features, indicating less shape variation. This demonstrates that the ion implantation method reduces unnecessary interfaces, leading to a higher success rate and yield in future mass production. Figure 2c,d shows the linear current‐voltage (IV) and semi‐logarithmic current density‐voltage (JV) characteristics of four different structures when the device sizes are 10 × 10 µm^2^ and 100 × 100 µm^2^ (The electrical properties for all sizes are detailed in Figures S2–S4, Supporting Information). The IV characteristics were analysed using Keysight B1500 semiconductor analyser. By comparing the devices obtained through mesa etching and ion implantation, it can be observed from Figure 2c that the ion‐implanted devices exhibit a larger current at the same voltage, indicating a smaller series resistance. Figure 2d shows that when reaching the same current density, the ion‐implanted devices require a lower driving voltage. The turn‐on voltages (measured at 10 A cm^−^^2^) for all device configurations range from 2.83 to 2.89 V, with ion‐implanted structures consistently showing slightly lower values (2.83–2.85 V) compared to mesa‐etched devices (2.86–2.89 V), Ion‐implanted devices exhibit lower turn‐on voltage than ICP mesa‐etched devices because ion implantation minimizes sidewall damage and preserves carrier transport efficiency, while mesa etching introduces defects that increase series resistance and hinder current injection. Simultaneously, benchmarking against alternative ion implantation schemes – including heavy ions (As+/Ar+),^[^ ^25^, ^33^ ^]^ and light ions (He⁺)^[^ ^34^ ^]^ – demonstrates our F^‐^‐implanted devices deliver superior performance: record‐low leakage current (≈1 × 10^‐^^11^ A at −5 V) while maintaining industry‐compatible turn‐on voltage (≈2.8 V). These results establish high‐energy F^‐^ implantation as the optimal pixel isolation strategy for micro‐LED arrays. Detailed comparison data and analysis are provided in Table S1 (Supporting Information). Additionally, regardless of the isolation method used, the electrical performance of the lateral and vertical structures does not differ significantly. This may be attributed to the fact that the n‐GaN homoepitaxial substrate is sufficiently thick to mitigate the effects of current crowding that typically occur in lateral structures.

**Figure 3:**
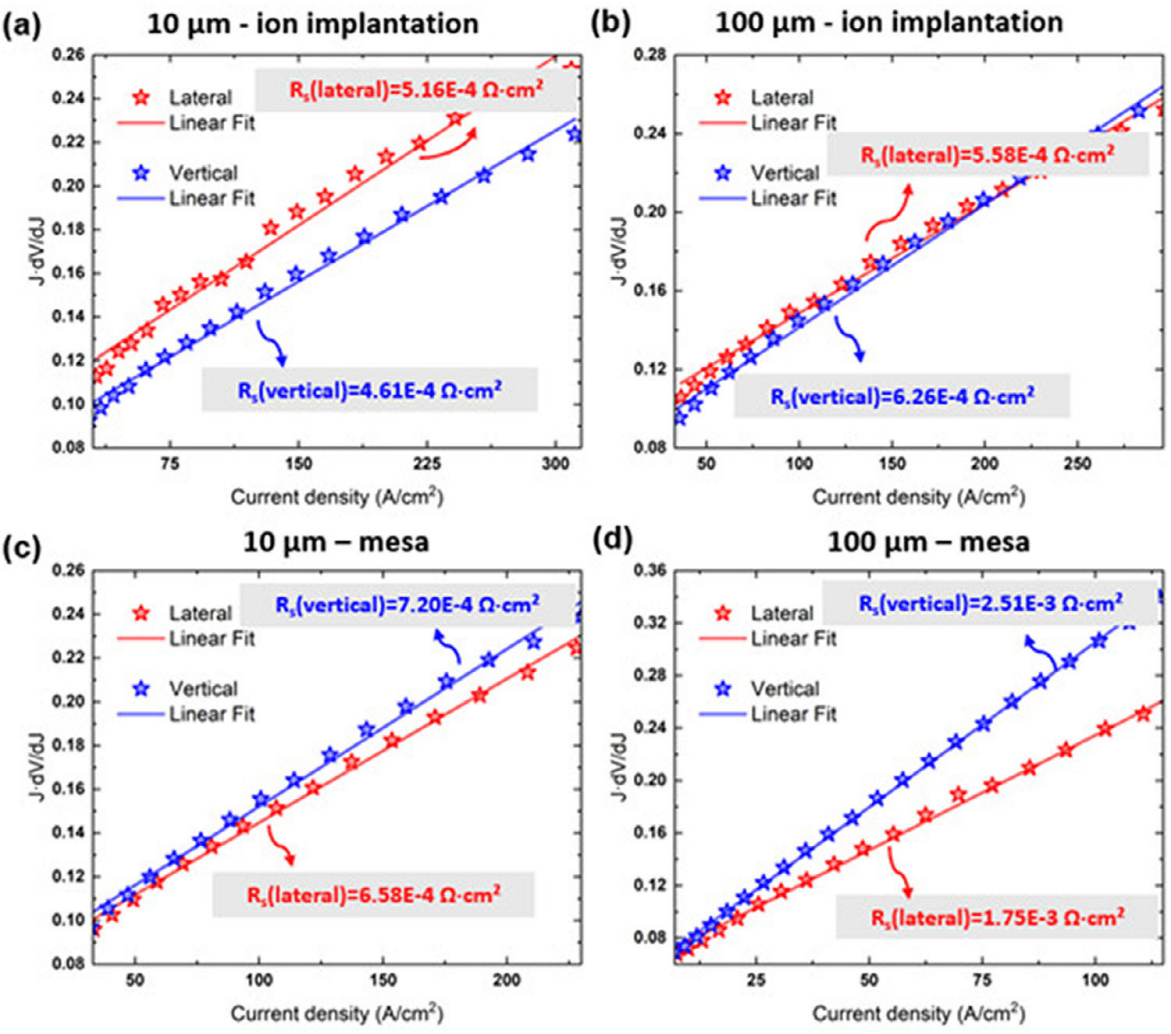
J × dV/dJ versus J curves for 10 × 10 µm^2^ a) and 100 × 100 µm^2^ b) devices using ion implantation, and for 10 × 100 µm^2^ c) and 100 × 100 µm^2^ d) devices using mesa etching.

The Ohmic series resistance can be extracted from the following formula, where J is the device current density, q is the electron charge, V is the applied voltage, R_S_ is the series resistance per unit area, n is the ideality factor of the diode, k is the Boltzmann constant, and T is the absolute temperature.

(1) $$J\frac{dV}{dJ}=JR_{S}+\frac{nkT}{q}$$

we can calculate the series resistance by plotting $J\frac{dV}{dJ}$ against J and extracting the slope of the linear relationship. The results, as shown in **Figure** **3**. By comparing Figure 3a,b, it is evident that devices with ion implantation structures exhibit smaller series resistance for smaller dimensions, which is consistent with the conclusions drawn from the IV curves. Although there are slight differences in series resistance between lateral and vertical structures, the variations are not significant. This observation also applies to devices with mesa ICP etching structures, as shown in Figure 3c,d. By comparing the series resistance of ion implantation structures with that of mesa structures (specifically comparing Figure 3a,c, as well as Figure 3b,d), it becomes clear that the series resistance of devices using ion implantation is significantly lower than that of traditional mesa structures. This difference is particularly pronounced in larger devices (100 x 100 µm^2^), where the resistance is only one‐fourth that of the mesa structure. This notable reduction in resistance may be attributed to the improved material quality provided by the ion implantation technique, which enhances device performance and effectively improves current conduction capability.

### 3.2 Optical Performance

**Figure 4:**
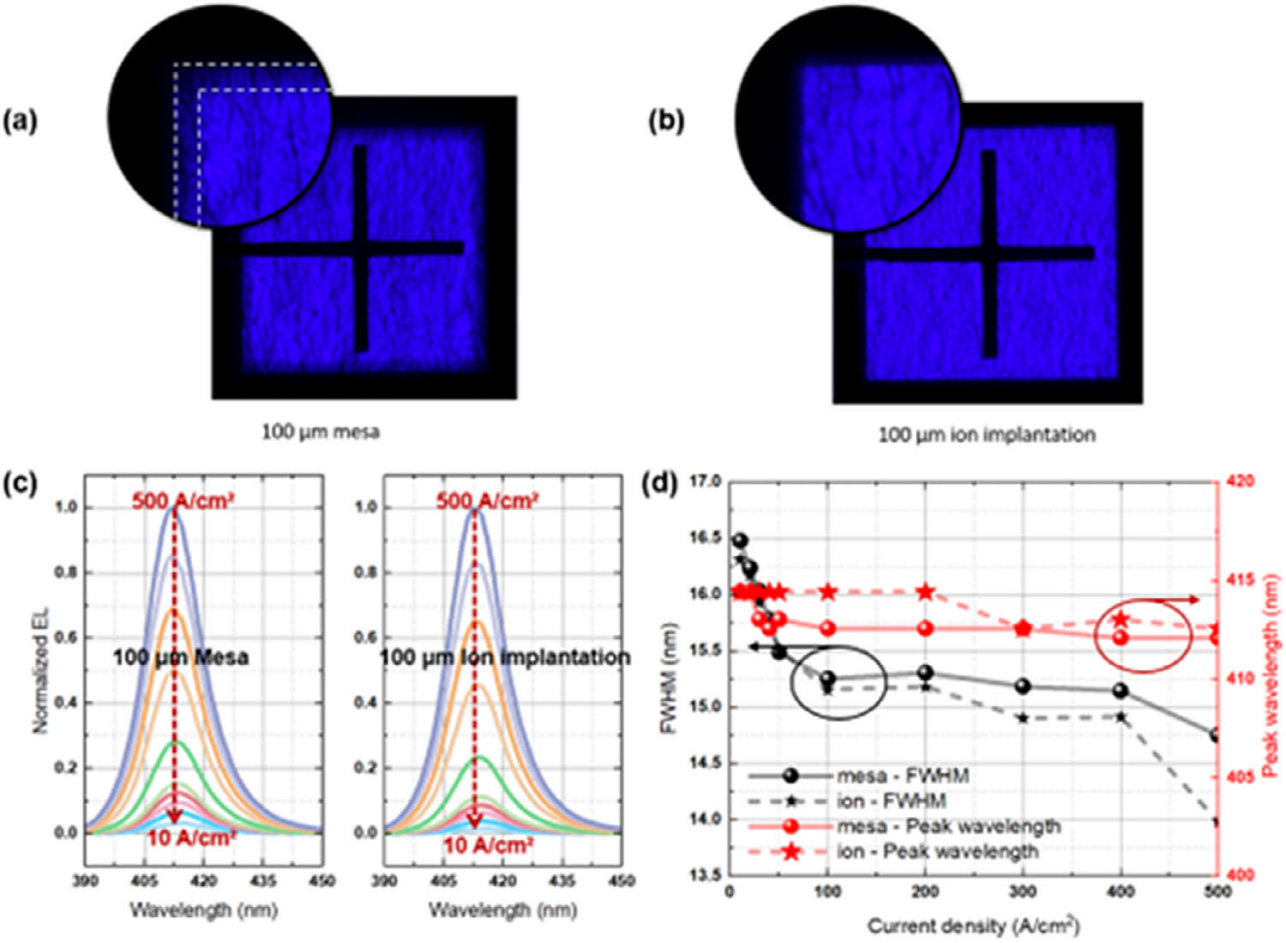
Electroluminescence images of 100 × 100 µm^2^ ion implantation (a) and mesa structures (b) at a driving current of 1 mA. (c) Normalized EL at different current densities (10, 20, 30, 40, 50, 100, 200, 300, 400, and 500 A cm^−^^2^), along with (d) the curves of FWHM and center wavelength as a function of current density.

**Figure** **4**a,b displays the electroluminescence (EL) images of ion and mesa structures (both sized at 100 × 100 µm^2^ and configured horizontally) at a driving current of 1 mA (Additional images are available in Figure S5, Supporting Information). Under the same driving conditions, the edges of the mesa structure device appear significantly darker than the center. This dimming is primarily due to the presence of more severe surface defects at the edges and sides, including dangling bonds and sidewall damage resulting from the ICP etching process. In contrast, the ion implantation method directly disrupts the lattice structure without creating side emissions. As a result, the edges of the ion devices are much clearer, exhibiting fewer surface defects and more uniform light emission. This uniformity in light output is crucial, as it can significantly enhance display resolution in future applications.

Figure 4c shows the spectra of the two structures at different current densities (the curves have been smoothed for clarity, with a smoothing window set to 8 in the original software). The use of ion implantation structures reduces non‐radiative recombination centers, resulting in a more rapid increase in light emission intensity as the current density rises from 10 to 500 A cm^−^^2^. Quantitative analysis reveals that the light intensity of the mesa‐etched structure increases from 0.033 to 1 (normalized) as the current density rises from 10 to 500 A cm^−^^2^, whereas the ion‐implanted structure shows a more pronounced enhancement from 0.015 to 1 under the same conditions. The ion‐implanted structure exhibits 117% higher light‐intensity growth versus mesa‐etched devices, attributable to its superior carrier confinement that suppresses efficiency droop. This quantifies the advantage of implantation‐based isolation for high‐current‐density operation. This also indicates that ion implantation structures perform better in thermal management, effectively dissipating heat and avoiding performance degradation due to thermal accumulation. The FWHM and center wavelength extracted from the spectra are shown in Figure 4d. As the current density increases, both types of devices exhibit relatively stable center wavelengths, approximately at 413 nm, while the FWHM gradually decreases from 16.5 to 14 nm. Due to the superior material quality achieved through ion implantation, its FWHM is smaller than that of the devices using mesa etching. The defects introduced by mesa etching can lead to non‐uniform light emission, which in turn increases the FWHM.

While the inherent Lambertian radiation pattern of LEDs is appealing for wide‐view television applications,^[^ ^35^ ^]^ it is less efficient for near‐eye display applications such as VR/AR. For personal electronic devices, users generally observe the display screen from a nearly horizontal angle as discussed earlier.^[^ ^36^ ^]^ Only the light emitted from the top of the pixel (within the escape cone) is beneficial for display purposes. Therefore, relying solely on EQE is insufficient to fully assess the light‐emitting performance. Even micro‐LEDs with high EQE can exhibit relatively low on‐axis intensity.^[^ ^37^ ^]^

**Figure 5:**
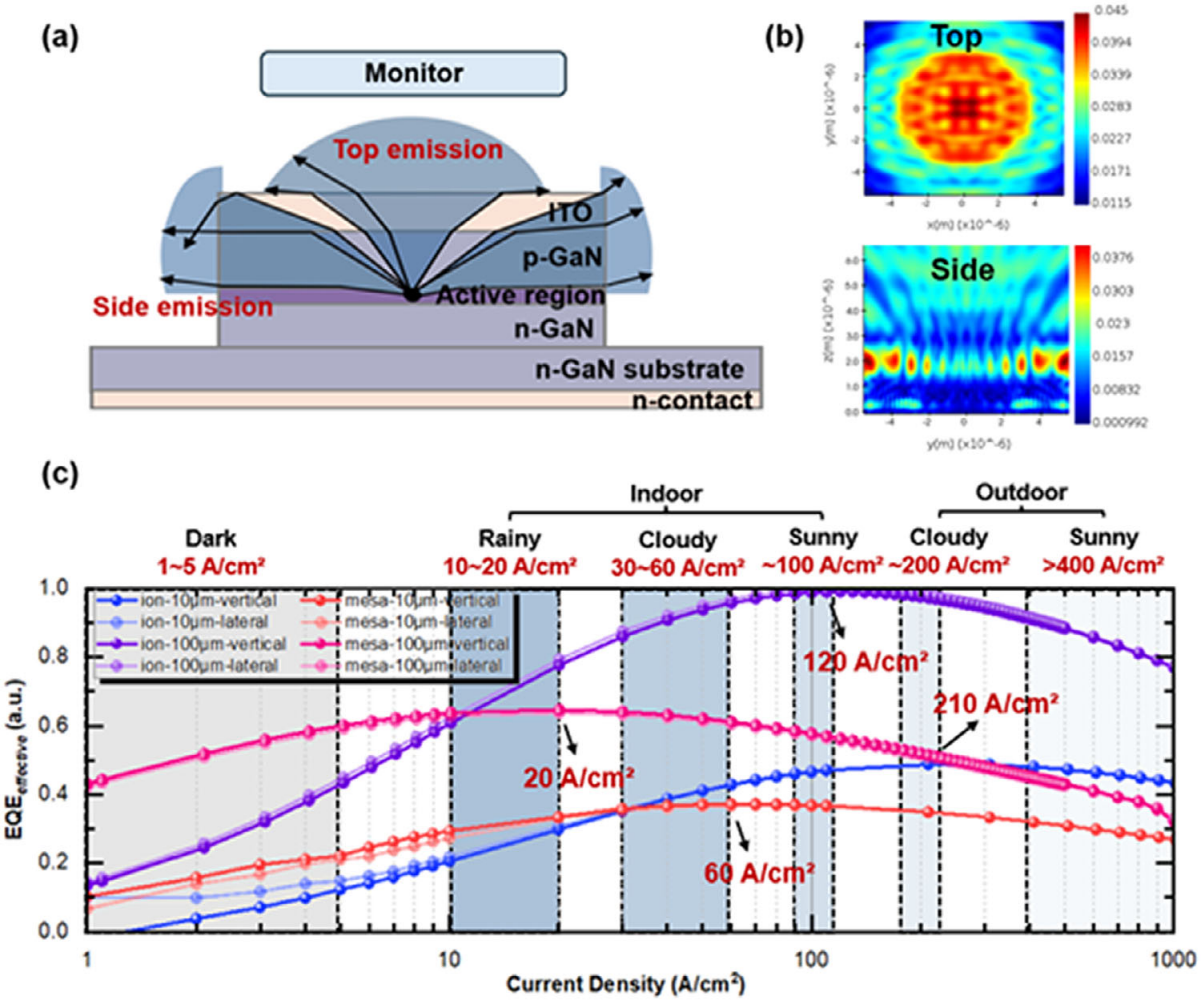
a) Schematic diagram of light distribution emitted from the mesa structure; b) Simulated top and side transmission power intensity distribution; c) Normalized display effective EQE of four structures at different current densities. The dashed colored boxes from left to right represent the working current density (A cm^−^^2^) required for micro‐LEDs as display sources in various AR glasses application scenarios. Data of working current density from.^[^ ^5^ ^]^

For mesa structures, as shown in **Figure** **5**a, the measured EQE includes contributions from both top emission and side emission (from the four sidewalls). However, the light emitted from the sides is not useful for near‐eye displays. Thus, we introduce the following formula to define the display effective EQE for mesa structures, where η_*top*_ is the emission efficiency (η defined as the ratio of transmitted power to source power) of the light emitted from the top,  η_*side*_is the emission efficiency of the light emitted from the four sidewalls of the mesa structure, and *EQE_all_* refers to the overall EQE measured using integrating sphere.

(2) $$EQE_{effective}=\frac{\eta_{top}}{\eta_{top}+\eta_{side}}EQE_{all}$$

Using Lumerical FDTD simulation, we set a single dipole in the MQW active region, and the height of the top monitor is set according to the escape cone angle (The structure of the FDTD simulation model is illustrated in Figure S6, Supporting Information), and the resulting top and side transmission power distributions are shown in Figure 5b. The top distribution indicates that the expected square shape has become rounded due to side emission, resulting in a less distinct outline. The sidewall distribution exhibits higher intensity at the edges, likely due to the combined effects of front and back light emission. Then we obtained η_*top*_ of 3.12% and η_*side*_ of 6.10%. This results in a top emission ratio of 33.87%. Combining this with *EQE_all_* data measured using the integrating sphere, we can calculate *EQE_effective_* for the mesa structure. Since the ion implantation structure only has top emission, its measured *EQE_all_* is equivalent to its *EQE_effective_*. The results are shown in Figure 5c. It is evident that both large (100 × 100 µm^2^) and small (10 × 10 µm^2^) ion implantation structures exhibit significantly higher display effective EQE values. The peak current densities (J_peak_) for the two sizes of mesa structures are 20 and 60 A cm^−^^2^, respectively, while the ion structures have J_peak_ values of 120 and 210 A cm^−^^2^, Both structures show reduced peak EQE and increased J_peak_ at smaller sizes, but the underlying mechanisms differ. In mesa‐etched µLEDs, the degradation is primarily attributed to enhanced non‐radiative recombination at sidewall.^[^ ^38^ ^]^ Conversely, in ion‐implanted µLEDs (lacking etched sidewalls), the size dependence stems from lateral carrier diffusion. Lateral carrier diffusion causes the carrier density to peak at the device center and decrease toward the periphery. Crucially, the distance carriers diffuse laterally is comparable across different sizes.^[^ ^39^ ^]^ Therefore, a larger fraction of carriers diffuses beyond the intended active area in smaller devices, recombining non‐radiatively via SRH (effectively increasing A) and lowering the average carrier density within the emitting region.^[^ ^39^ ^]^ According to the ABC model (R = An + Bn^2^ + Cn^3^), this reduced density favors non‐radiative SRH recombination (An) over radiative recombination (Bn^2^). Consequently, higher current densities (J_peak_) are required to achieve peak EQE in smaller ion‐implanted devices, while the increased SRH losses lower the maximum EQE. Overall, the ion‐implanted structures exhibit both higher peak EQE and corresponding J_peak_ values across all sizes compared to mesa‐etched devices. This superior performance fundamentally stems from the elimination of plasma‐induced sidewall damage in the ion implantation process. Despite the lateral carrier diffusion in ion‐implanted devices, their active regions maintain significantly higher material quality. This enables more efficient radiative recombination across all sizes and more uniform carrier distribution effectively suppressing Auger recombination (C), resulting in consistently higher peak EQE and J_peak_ than mesa‐etched devices. Notably, in our previous work,^[^ ^5^ ^]^ we discussed that for near‐eye display devices like AR, the actual working current density must exceed 100 A/cm^2^ in outdoor or well‐lit indoor environments to achieve optimal viewing. This aligns with the peak values of the ion implantation structures, suggesting that they can maintain higher brightness levels at elevated current densities in practical applications. Using the same approach, with the integrating sphere and simulation results, we demonstrate that the ion‐implanted structure improves the wall‐plug efficiency (WPE) due to the reduced series resistance, as shown in Figure S7 (Supporting Information)—consistent with the electrical analysis results.

## 4 Conclusion

The pursuit of high‐brightness, high‐resolution, and compact near‐eye displays demands disruptive innovations in micro‐LED technology. In this work, we demonstrate that GaN‐on‐GaN homoepitaxial micro‐LEDs, when synergized with fluorine ion implantation for pixel isolation and vertical device architectures, offer a transformative solution. Replacing conventional mesa etching with ion implantation dramatically reduces series resistance while delivering optically superior devices—exhibiting stable emission wavelengths, narrowed FWHM, and pixel‐edge sharpness critical for resolution enhancement. The proposed display‐effective EQE metric further validates that ion‐implanted structures outperform traditional designs under practical operating conditions, particularly in high‐current‐density scenarios essential for AR/VR applications. Furthermore, vertical micro‐LEDs achieve a compact footprint without compromising electrical or optical performance, enabling ultra‐dense pixel integration. By unifying material advantages, advanced pixelation methods, and structural innovation, this work establishes a scalable framework for next‐generation micro‐LED displays, paving the way for immersive near‐eye systems with unprecedented pixel density and energy efficiency.

## Conflict of Interest

The authors declare no conflict of interest.

## Author Contributions

Z.L. and Y.L. contributed equally to this work. Conceptualization performed by Z.L., M.W., H.S.K.; Methodology performed by Z.L., Y.L., F.F., F.Y., M.T., Z.L., M.W., H.S.K.; Investigation performed by Z.L., Y.L., F.F., J.Z., S.H.; Visualization performed by Z.L. and Y.L.; Simulation performed by H.J.; Funding acquisition performed by Z.L., M.W., and H.S.K.; Project administration performed by Z.L., M.W., H.S.K.; Supervision performed by Z.L., M.W.; Writing – original draft performed by Z.L.; Writing – review & editing performed by Z.L. and M.W.

## Supporting information



Supporting Information

## Data Availability

The data that support the findings of this study are available from the corresponding author upon reasonable request.
