# Supplementary material for: Vertical GaN‐On‐GaN Micro‐LEDs for Near‐Eye Displays
Source: Adv Sci (Weinh). 2025 Jul 30;12(40):e06784. doi: 10.1002/advs.202506784 (PMC12561280; doi:10.1002/advs.202506784)
Supplement: Supplementary file 1 — Supporting Information [file ADVS-12-e06784-s001.docx]

**Supplementary Information**

Vertical GaN-on-GaN Micro-LEDs for Near-Eye Displays

Zichun Li ^1^, Yibo Liu^1^, Haonan Jiang^1^, Feng Feng^1^, Jingyang Zhang^1^, Shan Huang^1^, Fion Yeung^1^, Manchun Tseng^1^, Man Wong^1^, Hoi Sing Kwok^1^, Zhaojun Liu^2^

**^1^Hong Kong University of Science and Technology, Hong Kong, China**

**^2^Southern University** **of Science and Technology, Shenzhen, China**


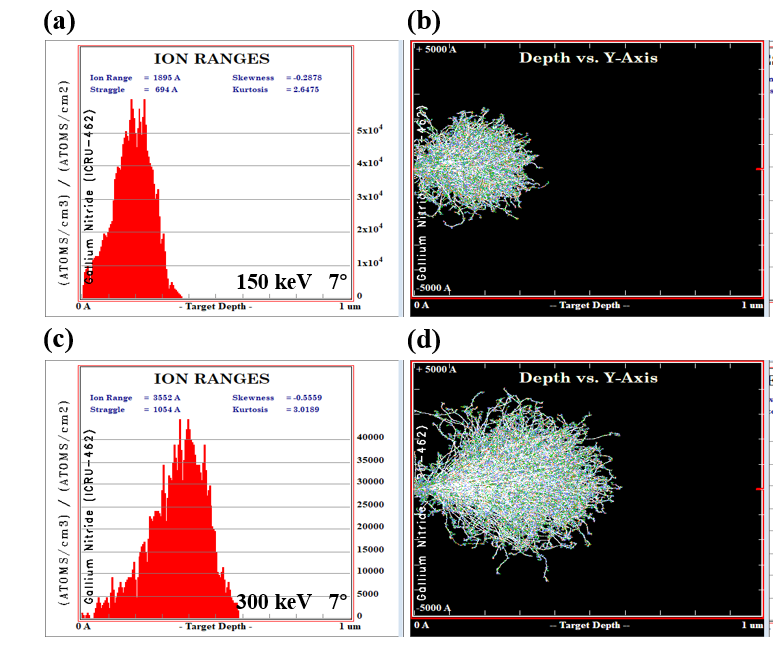


Fig.S1. SRIM simulation results of Fluorine ion implantation into GaN under different energy conditions. (a) and (b) show the ion concentration profile and trajectory distribution, respectively, for implantation at 150 keV and 7° tilt angle. (c) and (d) present the corresponding profiles for implantation at 300 keV with the same tilt angle of 7°.

We investigated the selection of ion implantation conditions using SRIM simulations, with the results shown in Fig.S1. As evidenced by SRIM simulations, increasing the ion implantation energy (e.g., 150 keV → 300 keV for Fluorine in GaN) extends the projected ion range but also amplifies lateral straggling due to nuclear/electronic scattering. Notably, experimental implantation depths may exceed simulated predictions, likely attributed to channeling effects (crystallographic alignment) or incomplete amorphization of the GaN lattice—factors not fully accounted for in SRIM’s binary collision approximation. In the final process, 150 keV implantation energy was selected for pixel isolation in GaN devices. This choice was experimentally validated to achieve effective electrical isolation between adjacent pixels, confirming that the ion-induced lattice amorphization depth sufficiently disrupts carrier transport paths while maintaining lateral confinement precision. The success at 150 keV—despite SRIM suggesting deeper penetration—likely reflects suppressed channeling effects through optimized tilt angles and the threshold defect density required for isolation in GaN-based systems.


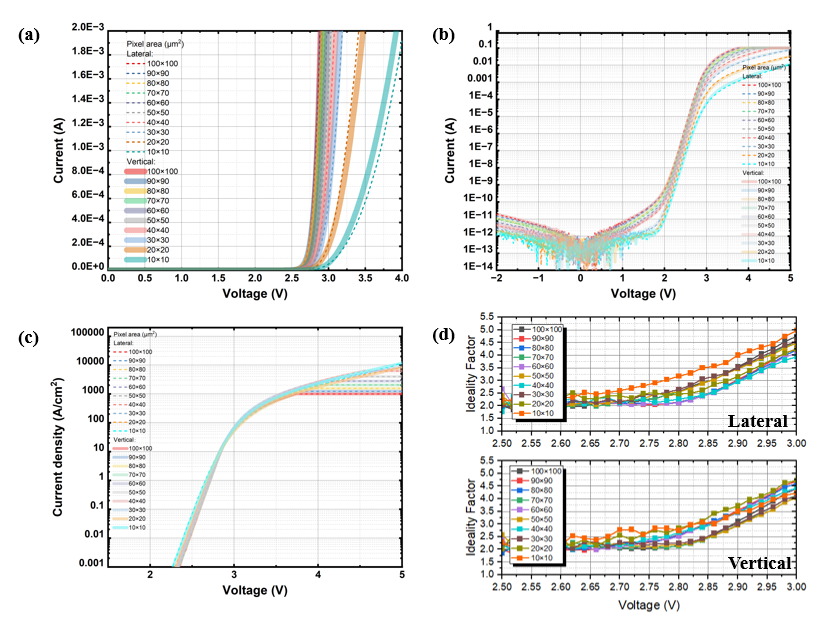


Fig.S2. Electrical characterization of pixel-isolated devices fabricated via ion implantation, featuring vertical and lateral configurations with pixel sizes ranging from 100×100 μm² to 10×10 μm². (a) Current-voltage (I-V) curves for devices of varying dimensions. (b) Corresponding I-V curves plotted on a logarithmic scale to highlight low-current behavior. (c) Current density-voltage (J-V) characteristics derived from the I-V data. (d) Extracted ideality factors for both vertical and lateral configurations.


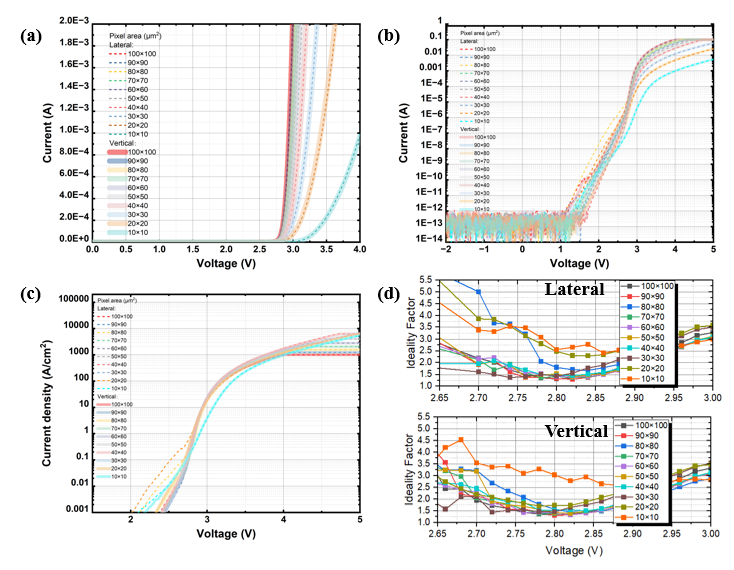


Fig.S3. Electrical characterization of pixel-isolated devices fabricated via conventional mesa ICP etching, featuring vertical and lateral configurations with pixel sizes ranging from 100×100 μm² to 10×10 μm². (a) Current-voltage (I-V) curves for devices of varying dimensions. (b) Corresponding I-V curves plotted on a logarithmic scale to resolve low-current regimes. (c) Current density-voltage (J-V) characteristics normalized by pixel area. (d) Extracted ideality factors for both vertical and lateral configurations


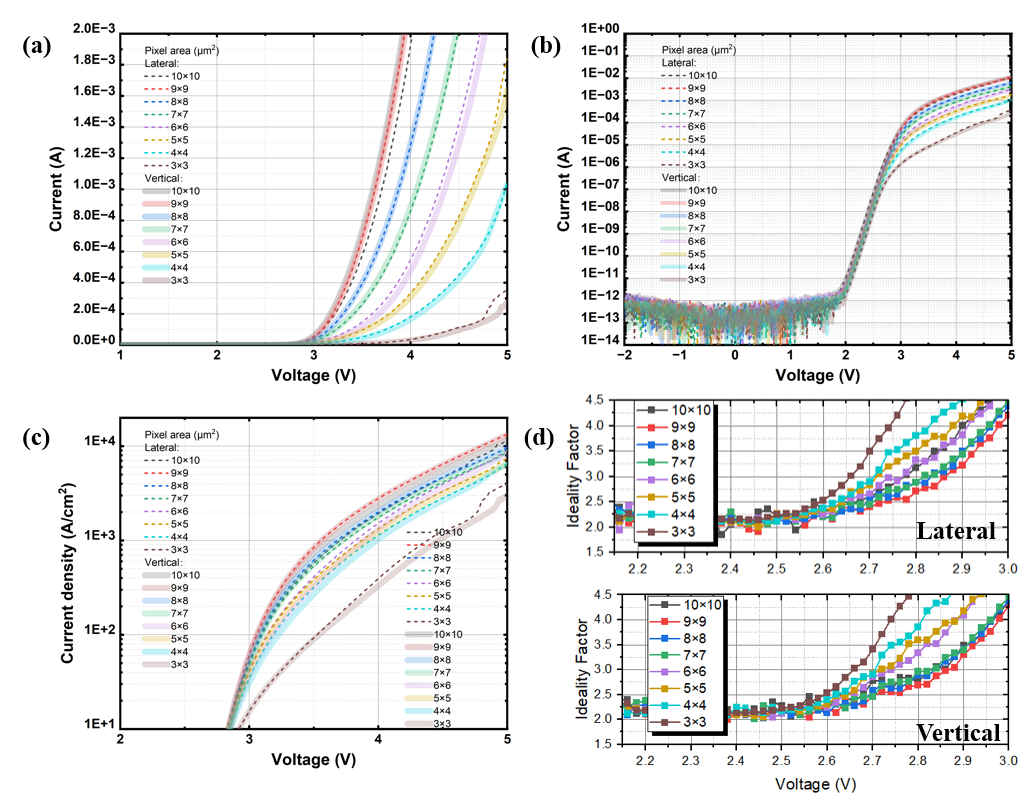


Fig.S4. Electrical characterization of ultra-small pixel-isolated devices (3×3–10×10 μm²) fabricated by ion implantation. (a) Size-dependent I-V curves for vertical/lateral configurations; (b) Log-scale I-V curves; (c) J-V characteristics; (d) Ideality factors contrasting vertical vs. lateral transport in sub-10 μm² pixels.

Fig.S2-S4 present the electrical characterization of devices fabricated via ion implantation and mesa ICP etching. Both methods exhibit excellent electrical performance, as evidenced by the I-V curves showing negligible leakage currents. The lateral and vertical configurations demonstrate comparable electrical characteristics, with minimal differences in forward/reverse bias behavior. Ideality factors, extracted using the modified Shockley equation (n = (q/kT)(dV/d(lnI))), reveal that ion-implanted structures achieve lower ideality factors compared to etched devices, suggesting reduced defect-assisted recombination at isolation interfaces.

Fig.S4 specifically highlights ion-implanted sub-10 μm² devices (down to 3×3 μm²), which retain excellent electrical integrity—low leakage and near-ideal I-V characteristics. Traditional mesa etched structures are omitted for sub-10 μm scales due to severe sidewall-dominated degradation: As pixel dimensions shrink, the increased sidewall-to-area ratio amplifies etching-induced sidewall damage (e.g., dangling bonds, surface states), leading to excessive leakage and catastrophic yield loss, rendering them impractical for nanoscale applications.


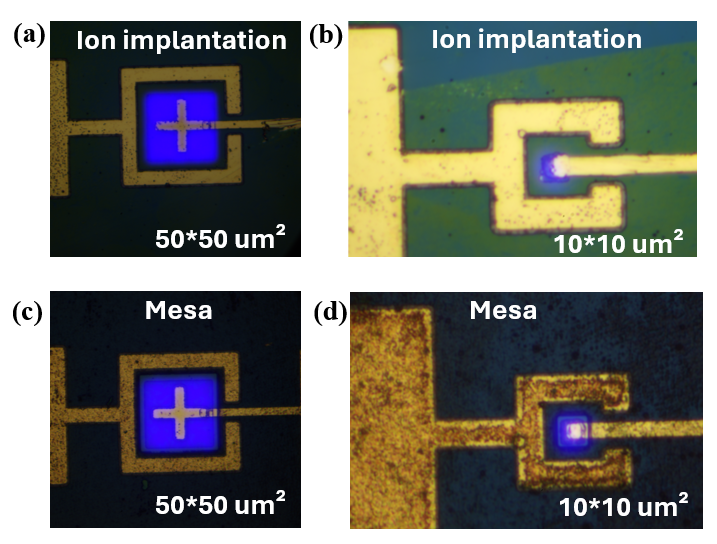


Fig.S5. Electroluminescence (EL) comparison of pixel-isolated devices: (a) 50×50 μm² and (b) 10×10 μm² devices fabricated via ion implantation; (c) 50×50 μm² and (d) 10×10 μm² devices using conventional mesa etching. The EL images highlight size-dependent luminescence uniformity and defect distribution, emphasizing the impact of isolation methodology on nanoscale optoelectronic performance.

**Table S1. Comparison of Ion Implantation Parameters and Device Performance**


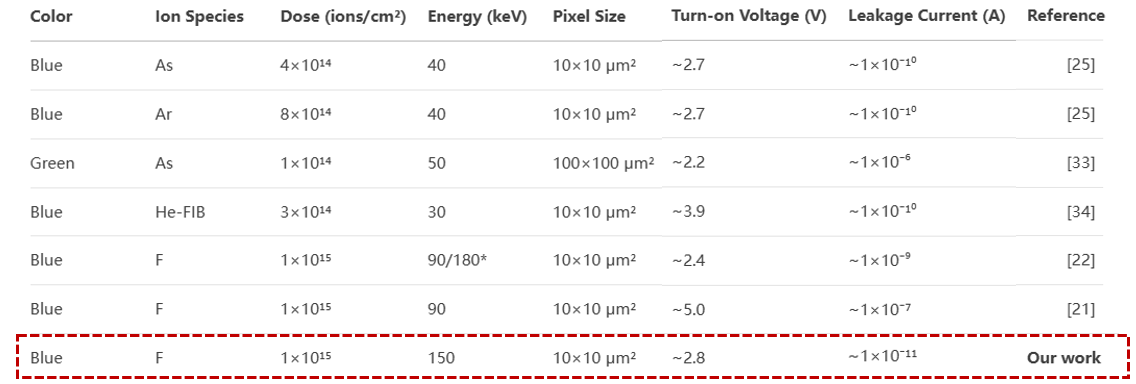


*Comparison of ion implantation parameters and electrical characteristics for different pixel configurations. Asterisk (*) indicates dual-energy implantation. Leakage currents measured at -5V.*

Our 150-keV F⁻ implanted devices achieve exceptionally low reverse leakage currents (∼10⁻¹¹ A) – the lowest among all compared works and effectively negligible for practical applications. This results from deep defect profiles generated by high-energy implantation, which create robust carrier blocking barriers between pixels. As evidenced by 2-4 orders of magnitude reduction versus other F⁻ implementations, this demonstrates optimal electrical isolation essential for high-density micro-LED arrays. While heavy ions (As/Ar) achieve low turn-on voltages (∼2.7V), they suffer from severe lateral straggling due to high nuclear stopping power, which may cause defects to spread beyond mask boundaries. This may induce pixel crosstalk and non-uniform emission. Light ions (He⁺/H⁺) exhibit shallow damage and low leakage, but their minimal lattice disruption enables rapid dynamic annealing during operation. This may lead to thermal instability above 100°C, degrading isolation quality during sustained mid-temperature operation – a critical limitation for industrial applications. All ion implantation approaches except light ions achieve comparable turn-on voltages within the optimal 2-3V range. This consistency confirms that medium-to-heavy ions preserve junction integrity regardless of species. The voltage elevation in light ions may stem from: a) Incomplete lattice amorphization creating carrier trapping sites; b) Dynamic annealing effects during operation degrading isolation quality.


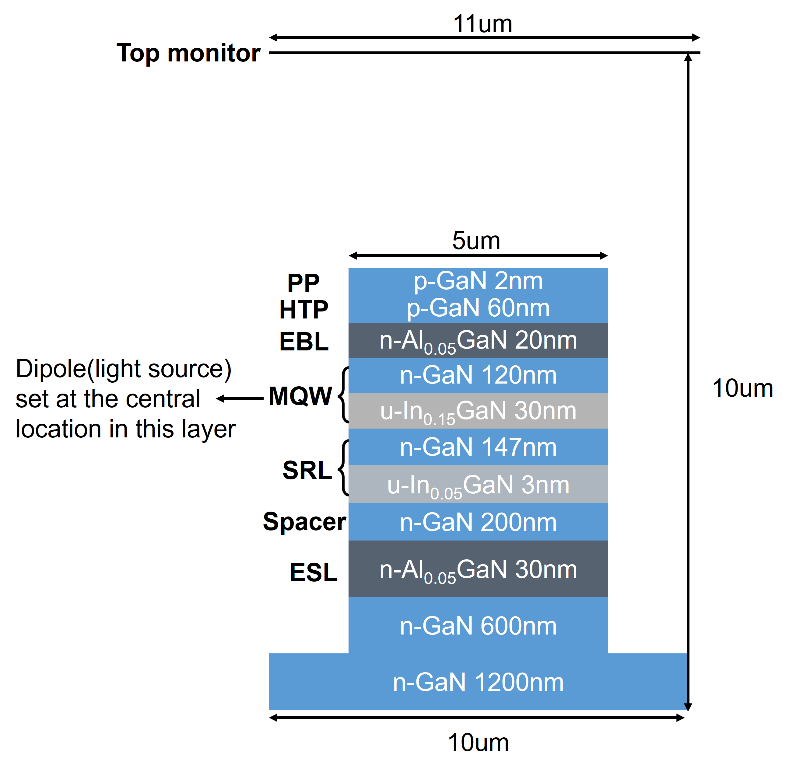


Fig.S6. FDTD simulation model schematic (Lumerical) with detailed layer parameters: Material stack thicknesses and monitor position.


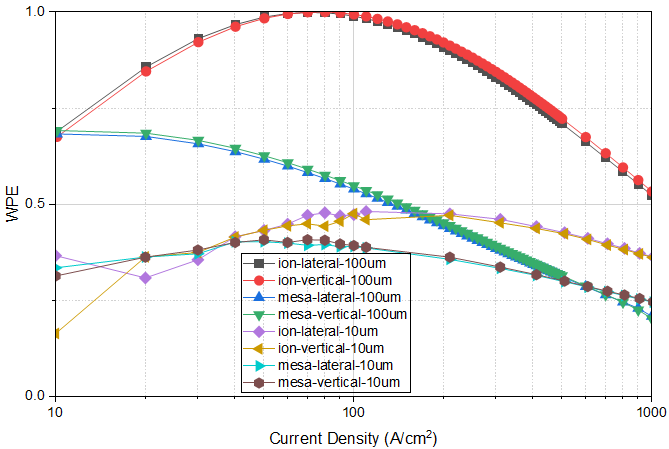


**Fig. S7. Normalized wall-plug efficiency (WPE) comparison between lateral and vertical structures in 10×10 and 100×100 μm^2^ pixels using two isolation methods.**
